# Modulation of host gene expression by the zinc finger antiviral protein

**DOI:** 10.1073/pnas.2420819122

**Published:** 2025-03-27

**Authors:** Daniel Gonçalves-Carneiro, Emily Mastrocola, Xiao Lei, Paul D. Bieniasz

**Affiliations:** ^a^Department of Infectious Disease, St Mary’s Medical School, Imperial College London, London W2 1NY, United Kingdom; ^b^Laboratory of Retrovirology, The Rockefeller University, New York, NY 10065; ^c^HHMI, The Rockefeller University, New York, NY 10065

**Keywords:** zinc finger antiviral protein, CpG dinucleotides, virus, mRNA

## Abstract

The zinc finger antiviral protein (ZAP) depletes nonself RNAs through recognition of their elevated CpG dinucleotide content. CpG dinucleotides are sparse in most endogenous mammalian mRNAs, but a subset might potentially be modulated by ZAP. While CpG frequency alone is insufficient to predict ZAP-regulation, we developed an algorithm using experimentally determined compositional features to predict which endogenous mRNAs may be ZAP-regulated. Using ZAP-knockout mice, we demonstrate that levels of many host mRNAs that are algorithmically predicted ZAP targets are indeed increased when ZAP is absent. ZAP is interferon-inducible, and we also identify genes that are downregulated by ZAP during an innate immune response. Many ZAP-regulated gene products are extracellular matrix or of nucleosome components, whose ZAP-mediated control is conserved in human cells. Overall, we provide a tool for the prediction of ZAP target genes and reveal host mRNAs that are ZAP-regulated.

## Significance

Many transcriptional and post-transcriptional mechanisms control gene expression in eukaryotic cells. The zinc finger antiviral protein (ZAP) is induced by interferon and inhibits viral gene expression and replication by recruiting a destructive nuclease to RNA sequences that are rich in CpG dinucleotides found in some viral genomes. However, the extent to which ZAP also impacts the expression of host genes is unclear. Using criteria delineated by measuring the ZAP effects on synthetic viral genomes, we designed an algorithm that predicts which host genes will be regulated by ZAP. Using ZAP-knockout mice, we show the accuracy of the algorithmic predictions and identify a small subset of host genes that are ZAP-regulated, particularly in the context of an interferon response.

The human genome exhibits extreme levels of CpG-suppression; that is a lower than expected number of CpG dinucleotides (1). The likely explanation for this phenomenon is that CpG dinucleotides have been purged over time. DNA methylation is important in epigenetic regulation of gene expression whereby DNA methyltransferases catalyze the conversion of cytosines, in a CpG dinucleotide context, to 5-methyl-cytosine (2). Methylated cytosines can undergo spontaneous deamination, generating C-to-T substitutions and consequent enrichment of TpG dinucleotides at the expense of CpG dinucleotides (3, 4). The resulting paucity of CpG dinucleotides in the genomes of humans and many other vertebrate species has created an opportunity for the emergence of host-defense genes whose products recognize CpG dinucleotides in exogenous nucleic acids, such as the Toll-like receptor 9 (5) and the zinc finger antiviral protein (ZAP) (6).

ZAP, encoded by the gene *ZC3HAV1*, is an RNA-binding protein that inhibits the replication of multiple viruses (7–10). ZAP recognizes CpG dinucleotides in viral RNA (6, 11, 12) and recruits the endonuclease KHNYN (13) which is likely responsible for target RNA removal. The gene *ZC3HAV1* encodes at least four ZAP isoforms, although so-called short (ZAP-S) and long (ZAP-L) isoforms are the most abundant (14). These two isoforms are reported to have different subcellular localization, which may impact their antiviral activity (15, 16). ZAP has also been suggested to both promote and inhibit the development of a variety of human cancers including pancreatic (17), colorectal (18), and liver cancers (19), but the mechanisms underlying these reported effects are not understood.

The low abundance of CpG dinucleotides in human genomes and transcripts suggests that most endogenous host genes should not be targeted by ZAP (20). Nevertheless, evidence for a role of ZAP in modulating the expression of endogenous genes has been reported in the HeLa cell line (21), and more recently in the human embryonic kidney 293 T cell line (22). These apparent regulatory properties of ZAP were not linked to CpG content in the modulated genes, even though CpG dinucleotides are clearly the preferred binding target of ZAP (6, 11, 12). In principle, ZAP could modulate host gene expression by directly targeting mRNAs, while changes in the levels of ZAP targets could have secondary effects on additional genes, depending on the identity of primary ZAP targets. Given the low frequency of CpG dinucleotides in the mammalian transcriptome, predicting which genes would be directly targeted by ZAP solely based on CpG frequency poses significant challenges. However, using synonymously recoded HIV-1 genomes as a model, we recently determined general “rules” that govern RNA recognition and depletion by ZAP (23). Herein, we developed an algorithm that designates a ZAP-sensitivity score to each gene of humans and mice, based on nucleotide compositional features. We test the accuracy of this prediction tool by comparing the transcriptomes of ZAP^+/+^ and ZAP^−/−^ mice, as well as normal and ZAP-deficient human cells. In so doing, we identify a set of genes whose expression is regulated by ZAP, likely across mammalian species. Our results provide tools with strong predictive power and reveal many endogenous targets of ZAP.

## Results

### Predicting ZAP-Target Sites in the Human Transcriptome.

**Fig. 1.**
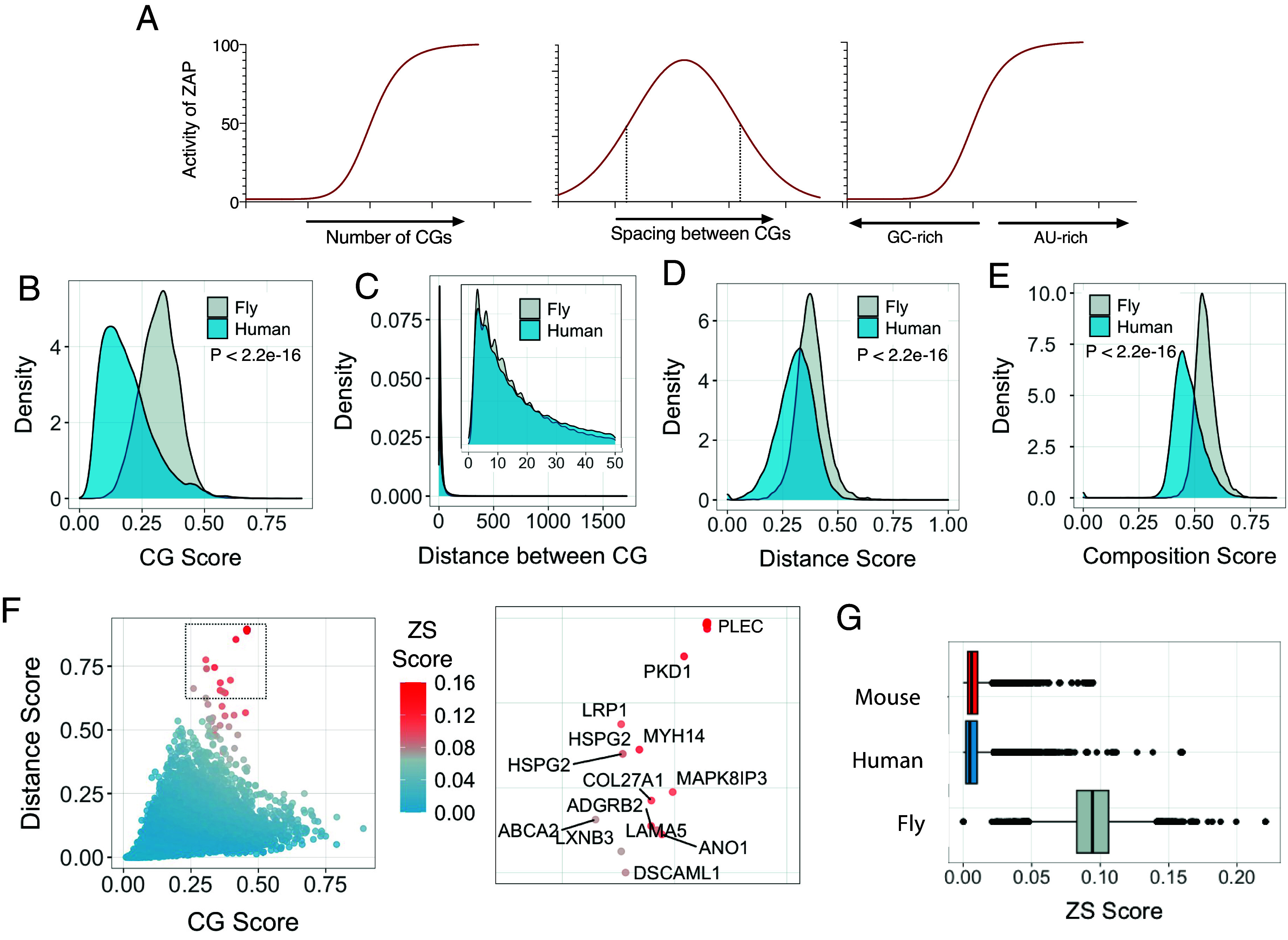
Predicting ZAP-target sites in the human transcriptome. (*A*) RNA features, specifically the number of CpG dinucleotides, spacing between CpG dinucleotides and AU-richness between optimally spaced CpG pairs, were used to calculate a ZAP-sensitivity score for each mRNA, as described in the *Methods*. (*B*–*E*) Kernel Distribution density estimates of CG scores (*B*), distance between CpG dinucleotides (*C*), distance scores (*D*), and composition scores (*E*) comparing human and Drosophila melanogaster (fly) protein-coding sequences, calculated as described in the *Methods.* (*F*) Distribution of ZAP-sensitivity (ZS) scores among human coding sequences. Expanded inset indicates the gene names of highly scored genes. (*G*) Distribution of ZS scores in coding sequences from the human (blue), mouse (red), and fly (gray).

Previously, we determined the features that contribute to optimal ZAP targeting in the context of the HIV-1 genome (23). Using these features, we were able to recode the genome of an unrelated virus to confer ZAP sensitivity. The three key features controlling ZAP sensitivity were the number of CpG dinucleotides present in the RNA target (which positively correlates with ZAP sensitivity), the spacing between CpG dinucleotides (with maximum ZAP sensitivity being achieved when CpG are located between 12 and 32 nucleotides from each other) and the mononucleotide composition of the sequence surrounding CpG dinucleotides (A/U-rich sequences conferred greater ZAP sensitivity) (Fig. 1*A*). Based on these features, we generated an algorithm that calculates three metrics associated with a given RNA molecule: 1) CG Score, which calculates the relative abundance of CpG dinucleotides in a molecule, 2) Distance Score, which calculates how many CpG pairs are optimally placed in a given mRNA transcript and 3) Composition Score, which assesses the A/U-richness of the sequences between CpG dinucleotides. These metrics were then combined to generate a single “ZAP-sensitivity” (ZS) score, ZS, (*Methods*) that predicts the relative ability of a particular RNA molecule to be targeted by ZAP.

We compiled an in silico database of human protein coding sequences and calculated the scores described above for each sequence. For comparison, we performed a similar analysis using a library of coding sequences from *Drosophila melanogaster* (fruit fly). We chose drosophila as a comparison organism because their genomes, and the genomes of insects in general, are less CpG-suppressed than human genomes (24). Moreover, ZAP emerged in ancestral tetrapods, while orthologs of ZAP are absent in insects (20), so any putative selective pressure that ZAP may have applied to the human genome is expected to be absent in insects. CG scores in *D. melanogaster* mRNAs were higher than in human sequences (Fig. 1*B*), as predicted, given that fly genomes are less CpG-suppressed than human counterparts. The overall distribution of the distances between CpG pairs was superficially similar in the two species (Fig. 1*C*). Nevertheless, drosophila coding sequences have, on average, smaller distance between CpG pairs (~16.8 nucleotides) when compared to human coding sequences (~29.9 nucleotides). Interestingly, in both species, the most frequent nucleotide distances between CpG motifs were multiples of three (Fig. 1*C*, *Inset*). This phenomenon, which has been previously reported (25), is also observed for other dinucleotides and is postulated to be a consequence of the triplet nature of codons as it is absent in noncoding sequences. However, CpG pairs that were optimally distant from each other for ZAP sensitization were more abundant in fly than in human sequences, perhaps as a result of selection pressure in human genomes (Fig. 1*D*). Additionally, the mononucleotide composition of sequences between CpG dinucleotides was, on average, more AU-rich in drosophila coding sequences than in human sequences (Fig. 1*E*). Together, these observations indicate that the human transcriptome contains fewer features that would be predicted to confer ZAP sensitivity than does the *D. melanogaster* transcriptome.

To predict potential ZAP target mRNAs we combined CG, Distance and Composition scores and calculated a ZAP-sensitivity (ZS) score for each human protein coding sequence (Fig. 1*F*). While most human sequences scored poorly, a group of highly scored genes segregated from the main population of sequences (indicated by the intensity of red color in Fig. 1*F*, inset), suggesting that such genes could be potentially targeted by ZAP. Genes such as *PLEC*, *LRP1*, *MYH14, HSPG2,* and *LTBP4* had especially high scores (*SI Appendix*, Fig. S1*A*). A similar analysis of protein coding sequences from drosophila revealed that ZS scores in this species were higher than their human counterparts (Fig. 1*G*). Indeed, the mean ZS score in drosophila coding sequences was equivalent to human coding sequences scoring at the ~99.9th percentile of human ZS scores. Overall, human mRNAs are predicted to be much less sensitive to ZAP than drosophila counterparts; however, this requires definitive experimentation.

Enrichment analysis of human genes with the highest ZS scores (the top 2.5% of scored genes) showed that they are primarily involved in morphogenesis, nucleosome assembly, and development processes. Conversely, human genes with the lowest ZS scores were enriched for those involved in keratinization and detection and defense against exogenous stimuli (*SI Appendix*, Fig. S1*B*). Network analysis showed that genes with high ZS-scores encode proteins involved in transcription regulation, are part of the nucleosome or are located extracellularly (*SI Appendix*, Fig. S1*C*). While coding sequence variation is restricted by codon usage, untranslated region (UTR) sequence is not, and 5′ or 3′ UTRs may contain additional ZAP targets and contribute to the modulation of gene expression (21). We therefore applied our algorithm to a library of full-length human transcripts containing 5′UTRs, coding sequences, and 3′UTRs. Full-length transcripts had lower CG scores than coding sequences. Because 5′ UTRs have, on average, higher CpG frequencies than coding sequences, while 3′ UTRs have lower CpG frequencies (20) the greater length of 3′UTRs reflects their greater impact on transcript CG scores. Conversely, transcripts and coding sequences had comparable Distance and Composition scores (*SI Appendix*, Fig. S1 *D*-*F*). The ZS scores of human transcripts displayed a similar distribution to human coding sequences (*SI Appendix*, Fig. S1*G*) and high ZS score transcripts again included genes such as *LRP1*, *PLEC,* and *HSPG2* (*SI Appendix*, Fig. S1*H*). Overall, these results suggest that while the majority of the genes in the human genome are not predicted to be targeted by ZAP, a small subset of genes predicted to be involved in transcription regulation and extracellular functions are more likely to be potential ZAP targets.

### Concordance of Predicted ZAP-Target Genes in the Human and Mouse Transcriptomes.

**Fig. 2.**
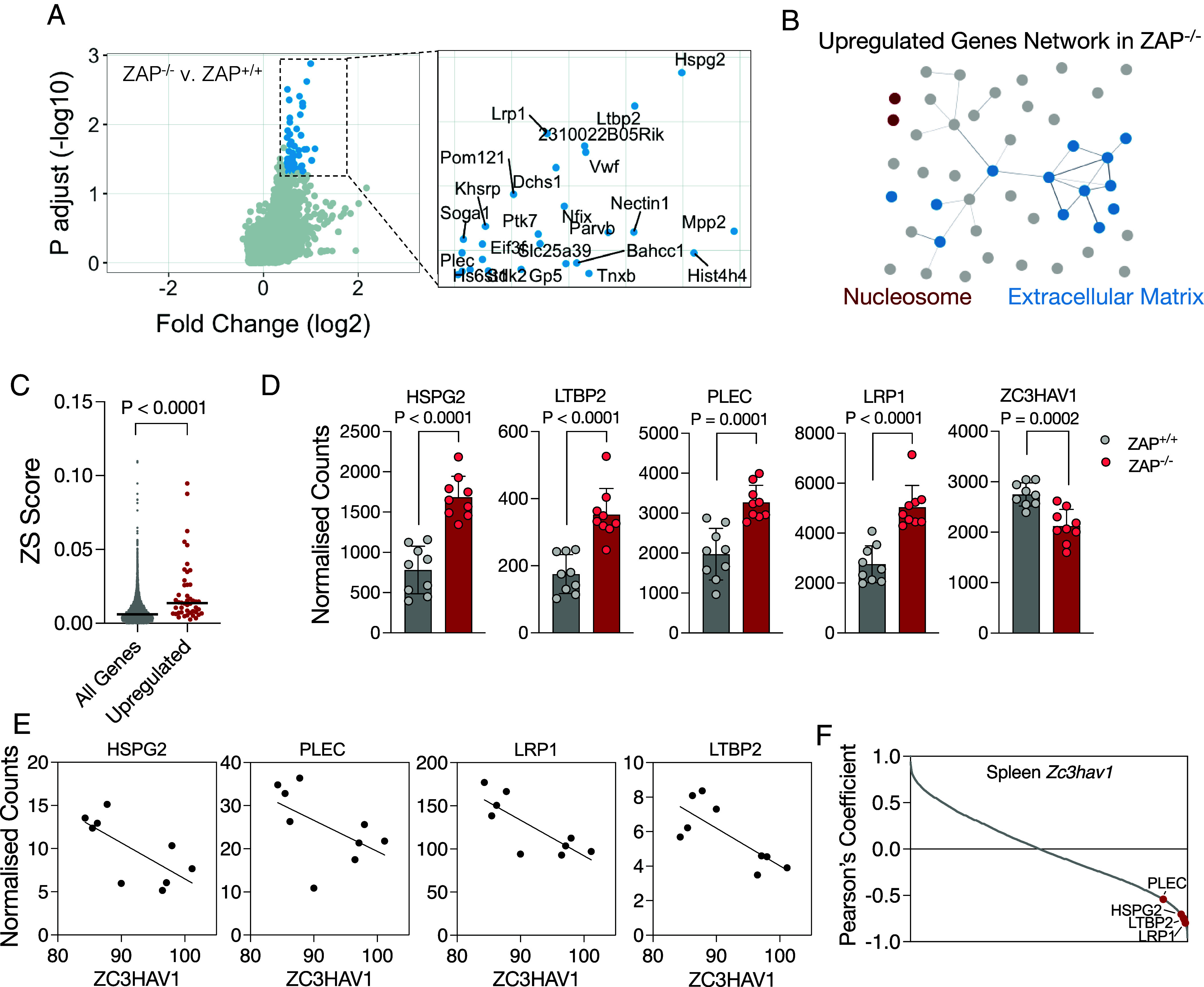
Differential gene expression in ZAP-deficient mice. (*A*) Transcriptomic (RNA-Seq) analysis of spleens from ZAP^+/+^ and ZAP^−/−^ mice. Blue dots and inset indicate genes more highly expressed in ZAP^−/−^ mice relative to ZAP^+/+^ mice. (*B*) Network analysis of upregulated genes in ZAP^−/−^ mice (*C*) Comparison of ZS scores for all mouse protein-coding sequences and the significant upregulated genes in ZAP^−/−^ mice identified in RNA-seq experiments. (*D*) Normalized RNA-seq counts of highly scored genes and *ZC3HAV1* (ZAP) in ZAP^+/+^ and ZAP^−/−^ mice (N = 9). (*E*) Linear regression analyses of the expression of *ZC3HAV1* (FPKM) and top scored genes in ZAP^+/+^ mice. (*F*) Pearson’s coefficient of correlation analyses performed between the expression of *ZC3HAV1* and of all other genes expressed in the spleens of ZAP^+/+^ mice. P, *P*-value.

ZAP orthologs are present in a variety of vertebrate species and mammalian zinc finger antiviral proteins have similar activity against CpG-rich RNA viruses (20). Mouse ZAP binds CpG dinucleotides in a similar manner to human ZAP (11, 12), suggesting that the mode of recognition and, therefore, optimal RNA targets should be an evolutionarily conserved trait in these two species. To test whether the mouse genome encoded mRNAs that could potentially be targeted by ZAP, we performed a similar analysis to that described above in which we calculated ZS scores for mouse coding sequences (*SI Appendix*, Fig. S2). Like human mRNAs, mouse mRNA sequences exhibited lower CG Scores, Distance Scores, and Composition Scores as compared to drosophila sequences (*SI Appendix*, Fig. S2 *A*–*C*). The distribution of mouse ZS scores exhibited a similar pattern to human sequences, with most sequences scoring poorly and a few genes exhibiting higher scores (Fig. 1*F* and *SI Appendix*, Fig. S2*D**)*. Many of these highly scored mouse genes – such as *Plec*, *Lrp1,* and *Hspg2* – were orthologues of highly scored human genes (*SI Appendix*, Figs. S1*A* and S2*E*), and an overall comparison of ZS scores between orthologous human and mouse genes, showed that the ZS scores of highly scored genes were correlated in the two species (*SI Appendix*, Fig. S2*F*). Conversely, lower ZS score values were less well correlated in the two species, which could be due to a threshold-like effect, whereby ZS scores below a certain value results in loss of control of expression by ZAP, making ZS score fluctuation inconsequential. In such a scenario, other selection pressures or drift would exert greater influence than ZAP binding on the evolution of these sequences. We also note an apparent trend toward marginally greater ZS scores for some human genes compared to their mouse counterparts (Fig. 2*F*), although the significance of this finding is unclear. Overall, these results suggest that coding sequences of a subset of murine and human mRNAs share features that are predicted to confer ZAP sensitivity.

### Differential Gene Expression in ZAP-Deficient Mice.

To evaluate whether ZAP could modulate endogenous gene expression in vivo, we first compared levels of mRNAs under normal, unstimulated conditions in wildtype and ZAP^−/−^ C57BL/6 mice whose derivation we have previously described (23). Spleens of ZAP^+/+^ or ZAP^−/−^ mice were harvested, total RNA was extracted and analyzed by RNA sequencing. In these comparisons, the levels of certain mRNAs were increased in mice lacking ZAP (Fig. 2*A*). Network analysis revealed that many mRNAs that were more abundant in ZAP^−/−^ mice encoded proteins involved in nucleosome assembly and extracellular matrix organization (Fig. 2*B*). Importantly, the ZS scores of the mRNAs that were more abundant in ZAP^−/−^ mice were higher than that of mouse genes in general (Fig. 2*C*). Among the top upregulated genes in ZAP^−/−^ mice were *HSPG2*, *LTBP2*, *PLEC,* and *LRP1* (Fig. 2*D*). Strikingly, expression of 87% of the genes with high ZS scores (the top 0.1% of scores using the ZS score algorithm) that were detectable by RNA-Seq, were increased in ZAP^−/−^ mice (*SI Appendix*, Fig. S3 *A* and *B*). To determine whether these genes were also increased in other tissues in the absence of ZAP, we analyzed total RNA extracted from lungs from ZAP^+/+^ and ZAP^−/−^ mice (*SI Appendix*, Fig. S3*C*). We found that *Lrp1*, *Ltbp4,* and *Plec* were also increased in the lungs of ZAP^−/−^ mice, albeit to a lesser extent compared to spleens. Because the levels of ZAP and its cofactors, TRIM25(26) and KHNYN(13), may dictate the extent to which ZAP-targeted genes are modulated, we compared the expression of *Zc3hav1*, *Trim25,* and *Khnyn* in the lung and spleen and, indeed, the levels of each of these mRNAs were lower in the lung than in the spleen (*SI Appendix*, Fig. S3*D*). Whole-organ proteomics data from mice (27) suggest a modestly higher level of ZAP (but not TRIM25 or KHNYN) in the mouse spleen as compared to the lung, while immunohistology studies of human tissues (28) also indicate that ZAP is more abundantly expressed in the spleen than in the lung (*SI Appendix*, Fig. S3 *E* and *F*). Consistent with a dose-dependent effect of ZAP, we found that the levels of *Zc3hav1* RNA in individual mouse spleens negatively correlated with the expression of *HSPG2*, *PLEC*, *LRP1,* and *LTBP2* (Fig. 2*E*). In fact, levels of these putative ZAP-regulated transcripts had the strongest negative correlation with ZAP levels among all genes expressed in the spleen (Fig. 2*F*). Together, these data suggest that the expression of genes that scored highly using our ZS algorithm is modulated by ZAP in mice.

### ZAP Modulates Gene Expression in Human Cells.

**Fig. 3.**
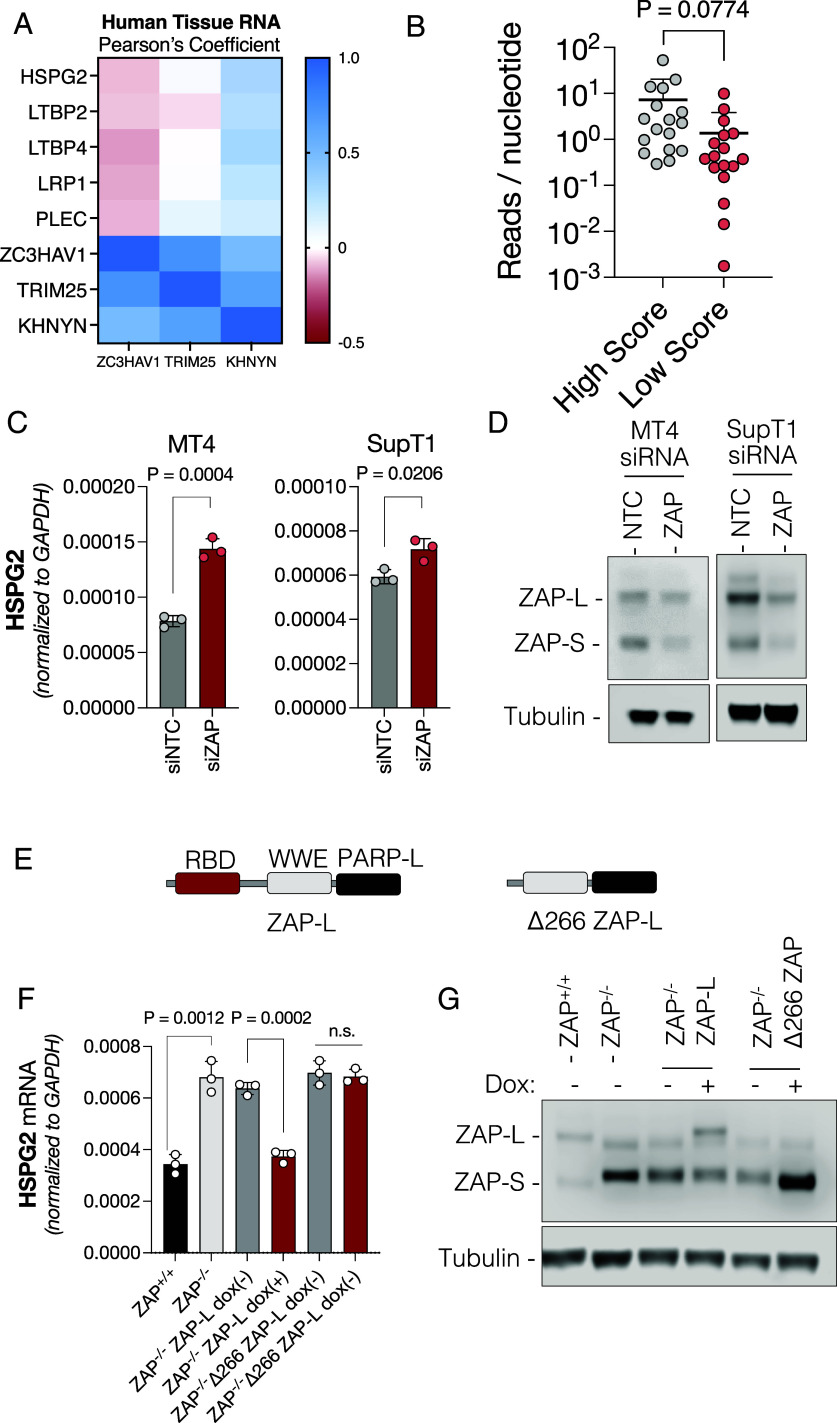
ZAP modulates gene expression in human lymphocytes. (*A*) Pearson’s coefficient of correlation analyses for the expression of *ZC3HAV1, TRIM25,* and *KHNYN,* compared to high ZS scored genes across human tissues. (*B*) Quantification of read density in ZAP CLIP-Seq experiments in MT4 cells for high ZS score and low ZS score genes. (*C*) *HSPG2* levels measured using qPCR in human T cell lines (MT4 and SupT1) transfected with siRNAs targeting ZAP or a nontarget control (NTC). (*D*) Western blot analysis of ZAP expression in transfected cells from panel (*C*). (*E*) Diagram of ZAP-L containing the RNA-binding domain (RBD), the WWE domain and the PARP-like (PARP-L) domain, and a truncated form of ZAP lacking the RBD (Δ266 ZAP-L). (*F*) *HSPG2* RNA levels in MT4 ZAP^−/−^ cells transduced with lentiviruses expressing ZAP-L or the Δ266 ZAP-L under the control of doxycycline (dox). RNA from cells treated with or without doxycycline was extracted and expression measured by qPCR. (*G*) Western blot analysis ZAP-L and Δ266 ZAP-L expression in cells treated with or without doxycycline. P, *P*-value.

To determine whether genes that were apparently modulated by ZAP in mice were also subject to ZAP regulation in human cells, we first performed a correlation analysis between *ZAP* mRNA levels and *HSPG2*, *LTBP2*, *LTBP4*, *LRP1,* and *PLEC* mRNA expression, using a public database of transcript abundance across all human tissues (28). Expression of these genes was negatively correlated with *ZAP/ZC3HAV1* levels (Fig. 3*A*), suggesting that these genes may also be modulated by ZAP in humans. Interestingly, the expression of KHNYN positively correlates with the expression of highly scored genes, for reasons that are unknown but might suggest coregulation of this ZAP cofactor and ZAP target genes. Using crosslinking immunoprecipitation coupled with RNA sequencing (CLIP-Seq), we found that ZAP bound profusely to numerous mRNAs in human MT4 cells, including *HSPG2* and *LTBP4* transcripts (*SI Appendix*, Fig. S4*A*). Indeed, we found that mRNAs of high ZS score genes were enriched in ZAP:RNA adducts isolated from human MT4 cells in CLIP-seq analysis, as compared to a control group of low ZS score human genes that were matched for both sequence length and transcript abundance in MT4 cells (Fig. 3*B* and *SI Appendix*, Fig. S4*B*). Together, these data indicate that the ZS score can predict the extent to which ZAP binds mRNAs in human cells.

To determine whether genes with high ZS scores were also sensitive to ZAP in human cells, we transfected human T lymphocyte cell lines with a pool of siRNAs targeting ZAP, extracted total RNA, and measured expression of two selected genes with similarly high ZS scores in mice and humans (*HSPG2* and *LTBP4*) by qPCR (Fig. 3 *C* and *D* and *SI Appendix*, Fig. S4 *C* and *D*). As was the case in the mouse spleen, reduction of ZAP expression in human T cell lines increased the levels of *HSPG2* and *LTBP4* mRNAs. To assess whether changes in gene expression were indeed dependent on ZAP, and specifically its RNA-binding activity, we transduced ZAP^−/−^ human MT4 cells with lentivirus vectors encoding a CRISPR-Cas9-resistant ZAP-L or a truncated derivative lacking the RBD (Δ266 ZAP-L) whose expression was controlled by the presence of doxycycline (Fig. 3*E*). While ZAP^−/−^ cells had higher levels of *HSPG2* mRNA than unmanipulated cells, induction of ZAP-L with doxycycline decreased *HSPG2* mRNA levels (Fig. 3 *F* and *G*). However, doxycycline induction of the truncated ZAP lacking the RBD did not impact *HSPG2* mRNA levels. In contrast, the expression of genes with low scores (such as *GNAS* and *RPS6KA1*) was not impacted by reconstitution with a functional ZAP-L (*SI Appendix*, Fig. S4*E*). Overall, these data show that ZAP can modulate the expression of genes with high ZS scores in human cell lines as well as in mouse spleens.

We applied our predictive model to genes that have previously been reported to be regulated by ZAP in other human cell lines (21, 22, 29). Todorova et al. found that the expression of TRAILR4 is modulated by ZAP. The transcript that encodes TRAILR4 (TNFRSF10D) has a ZS score at the 94.44th percentile and its levels are increased in human cells depleted of ZAP (*SI Appendix*, Fig. S4*F*). There is no known mouse orthologue of this gene, so we were not able to study this gene in our ZAP-knockout mice. Shaw et al. reported additional ZAP-regulated genes, including ADGRA3, FAM171A1, CERK, UNKL, DUSP7, SMTN, KDM6B, and CAMKK1. The degree to which these genes are differentially expressed in ZAP^−/−^ mice correlated with their ZS score (*SI Appendix*, Fig. S4*G*). Similarly, Ly et al. found genes that were apparently regulated by ZAP, which included MYEF2, TPM2, TIMP3, COL4A1, NT5DC2, SEC31A, LGALS3BP, CERK, CRISPLD1, HSPA8, BNIP3. The magnitude of the increases in the levels of these transcripts in ZAP^−/−^ compared to ZAP^+/+^ mice correlated with their ZS score (*SI Appendix*, Fig. S4*H*). While these genes do not correspond to those with the highest ZS scores, the degree to which they are upregulated in ZAP^−/−^ mice correlates with their ZS score. Further analysis of the interferon-repressed gene (IRG) dataset from Shaw et al. reveals that IRGs have higher ZS scores that interferon-stimulated genes (ISGs) (*SI Appendix*, Fig. S4*I*). The highest ZS score was attributed to the gene CERK, which both Shaw et al. and Ly et al reported to be regulated by ZAP. In our mouse dataset, CERK is more abundant in ZAP^−/−^ mice than wildtype mice (*SI Appendix*, Fig. S4*J*) consistent with their observations. Interestingly, we observed that HELZ2, an ISG, is unusual among ISGs in exhibiting a high ZS score (*SI Appendix*, Fig. S4*I*). While we observed induction of HELZ2 upon poly I:C treatment, HELZ2 expression was higher in ZAP^−/−^ mice (*SI Appendix*, Fig. S4*K*). This suggests that ZAP attenuates the increase of HELZ2 expression after the induction of an immune response. Overall, these analyses validate the ZS score algorithm, as it predicts genes regulated by ZAP across multiple studies and contexts.

### Interferon Transcripts Persist at Higher Levels in ZAP^−/−^ Mice Despite CpG Paucity.

**Fig. 4.**
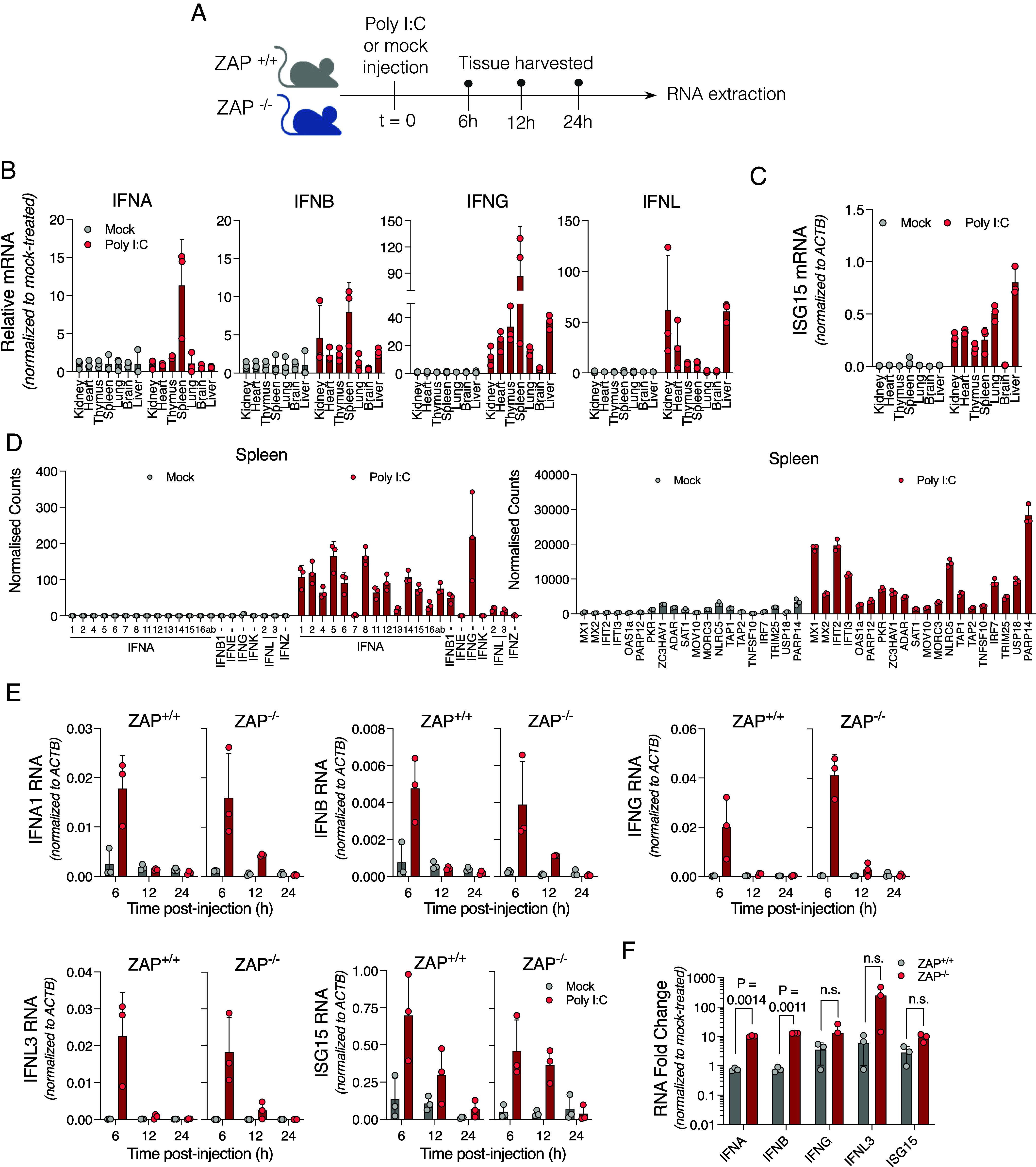
Interferon transcripts persist at higher levels in ZAP^−/−^ mice despite CpG paucity. (*A*) Experimental procedure used in transcriptomics experiments. ZAP^+/+^ and ZAP^−/−^ mice were injected intraperitoneally with poly I:C conjugated to JetPEI or a control solution containing only JetPEI. Organs were harvested 6, 12, and 24 h after injection, RNA was extracted and prepared for RNA-seq (N = 3 per group). (*B* and *C*) Expression of *IFNA*, *IFNB*, *IFNG*, *IFNL,* and *ISG15* measured by qPCR in the kidney, heart, thymus, spleen, lung, brain, and liver of mock-treated and poly I:C-treated ZAP^+/+^ and ZAP^−/−^ mice at 6 h post-injection. (*D*) Normalized RNA-seq counts of interferon transcripts (*Left*) and multiple ISG transcripts (*Right*) measured in mouse spleens at 6 h after poly I:C-treatment by RNA-Seq. (*E*) Expression of *IFN* and *ISG15* transcripts measured by qPCR in spleens from mock-treated or poly I:C-treated mice harvested 6, 12, or 24 h post-injection. (*F*) Comparison of fold changes in *IFNA*, *IFNB*, *IFNG*, *IFNL,* and *ISG15* RNA abundance in ZAP^+/+^ and ZAP^−/−^ mice treated with poly I:C at 12 h postinjection. P, *P*-value.

Even though ZAP is constitutively expressed (28), it is also upregulated upon interferon stimulation (30). Therefore, the putative gene modulatory effects of ZAP might be exacerbated during an immune response or inflammation. In fact, based on work in cell lines it has been proposed that the nucleotide composition of ISGs has been selected in order to evade the activity of ZAP during the innate immune response (29). To assess the effect of ZAP on IFN responses in vivo, we first established baseline conditions by injecting ZAP^+/+^ C57BL/6 mice with poly I:C intraperitoneally and harvested RNA from spleens at 2 h and 6 h postinjection. *IFNA1* and *IFNB1* transcripts were detected in spleens only when mice were injected with poly I:C complexed with a transfection reagent (*SI Appendix*, Fig. S5*A*). We next assessed IFN expression in seven whole organs including the kidney, heart, thymus, spleen, lung, and liver, by using qPCR to measure transcripts encoding IFN-α, IFN-β, IFN-γ, and IFN-λ following poly I:C injection with transfection reagent (Fig. 4 *A* and *B*). While *IFNA*, *IFNB,* and *IFNG* transcripts were most strongly induced in the spleen, *IFNL* transcripts were most potently induced in the kidney and the liver. In contrast to the rather tissue-restricted expression patterns of IFN transcripts following poly I:C administration, *ISG15* and *ZC3HAV1*, two ISGs, were broadly induced with elevated mRNA levels in all organs except the brain (Fig. 4*C* and *SI Appendix*, Fig. S5 *B* and *C*). Transcripts encoding many IFN-α variants as well as *IFNB1* and *IFNG* were elevated in the spleen after poly I:C treatment, as were transcripts encoding many ISGs and other proinflammatory cytokines (Fig. 4*D* and *SI Appendix*, Fig. S5*D*). Taken together, these data suggest that the administration of poly I:C via intraperitoneal injection leads to the expression of IFN transcripts in a tissue-dependent manner, presumably reflecting poly I:C transfection efficiency and sensor distribution, but increased ZAP and ISGs expression in multiple organs.

Previously it was reported that ZAP can affect the longevity of IFN transcripts in cell lines (31). We measured IFN transcripts in the spleens of poly I:C injected mice at 6, 12, and 24 post-injections (Fig. 4*E*). While transcripts encoding IFN-α and IFN-β were detected at comparable levels in ZAP^+/+^ and ZAP^−/−^ mice at 6 h post-injection with poly I:C, IFN-α, and IFN-β mRNA levels had declined at 12 h postinfection but were significantly higher in ZAP^−/−^ mice than in ZAP^+/+^ mice at that time point (Fig. 4 *E* and *F*). Levels of *IFNG* and *IFNL3* transcripts were also increased in ZAP^−/−^ mice at 12 h after injection, although this finding did not reach statistical significance. Despite the comparatively sustained levels of expression of *IFNA* and *IFNB* transcripts in ZAP^−/−^ mice compared to ZAP^+/+^ mice, ISG15 expression, as well as the expression of other ISGs, was not affected by ZAP, even at later time points (12 and 24 h) (Fig. 4*F* and *SI Appendix*, Fig. S5*E*). This is likely because the levels of IFN transcripts were substantially diminished compared to their peak at 6 h post-injection in both in ZAP^−/−^ and ZAP^+/+^ mice.

While these data suggested that the persistence of IFN transcripts may be reduced by ZAP, IFN transcripts have an extreme paucity of CpG dinucleotides and are among the genes with the lowest ZS scores in humans and in mice (*SI Appendix*, Fig. S5*F*). Nevertheless, many targets of ZAP are mRNAs that encode histones and transcription factors, that are naturally rich in CpG dinucleotides (6). Thus ZAP may downregulate transcription activators and inhibitors as well as histones, indirectly affecting the extent to which IFN and other genes are transcribed or posttranscriptionally regulated (32). Indeed, ZAP^−/−^ mice exhibited increased expression of many transcriptional repressors and chromatin modulators with comparatively high ZS scores, such as SPEN, ZFHX3, and NCOR2. Additionally, RC3H1 and RC3H2 are two RNA-binding proteins that limit the expression of many pro-inflammatory genes, including interleukin-6 (IL-6) and tumor necrosis factor (TNF), as well as many components of the NF-κB signaling cascade (33–36). We found that, in ZAP^−/−^ mice, both RC3H1 and RC3H2 are expressed at lower levels than in ZAP^+/+^ mice (*SI Appendix*, Fig. S5*G*), While it is conceivable that ZAP might directly stabilize RC3H1 and RC3H2 transcripts, more likely is a scenario in which changes in RC3H1 and RC3H2 levels represent secondary or tertiary effects of ZAP on gene expression given the aforementioned effects on histones and transcription factors. The apparent increase of IFN mRNA levels in ZAP^−/−^ mice is thus likely due to the ZAP-dependent regulation of genes involved in the synthesis or turnover of IFN mRNAs. Importantly, however, the magnitude of this effect was insufficient to impact the levels or duration of canonical ISG expression in response to poly I:C in mice.

### ZAP Down-Regulates Host Genes with Higher ZS Scores at Later Stages of the Innate Immune Response.

**Fig. 5.**
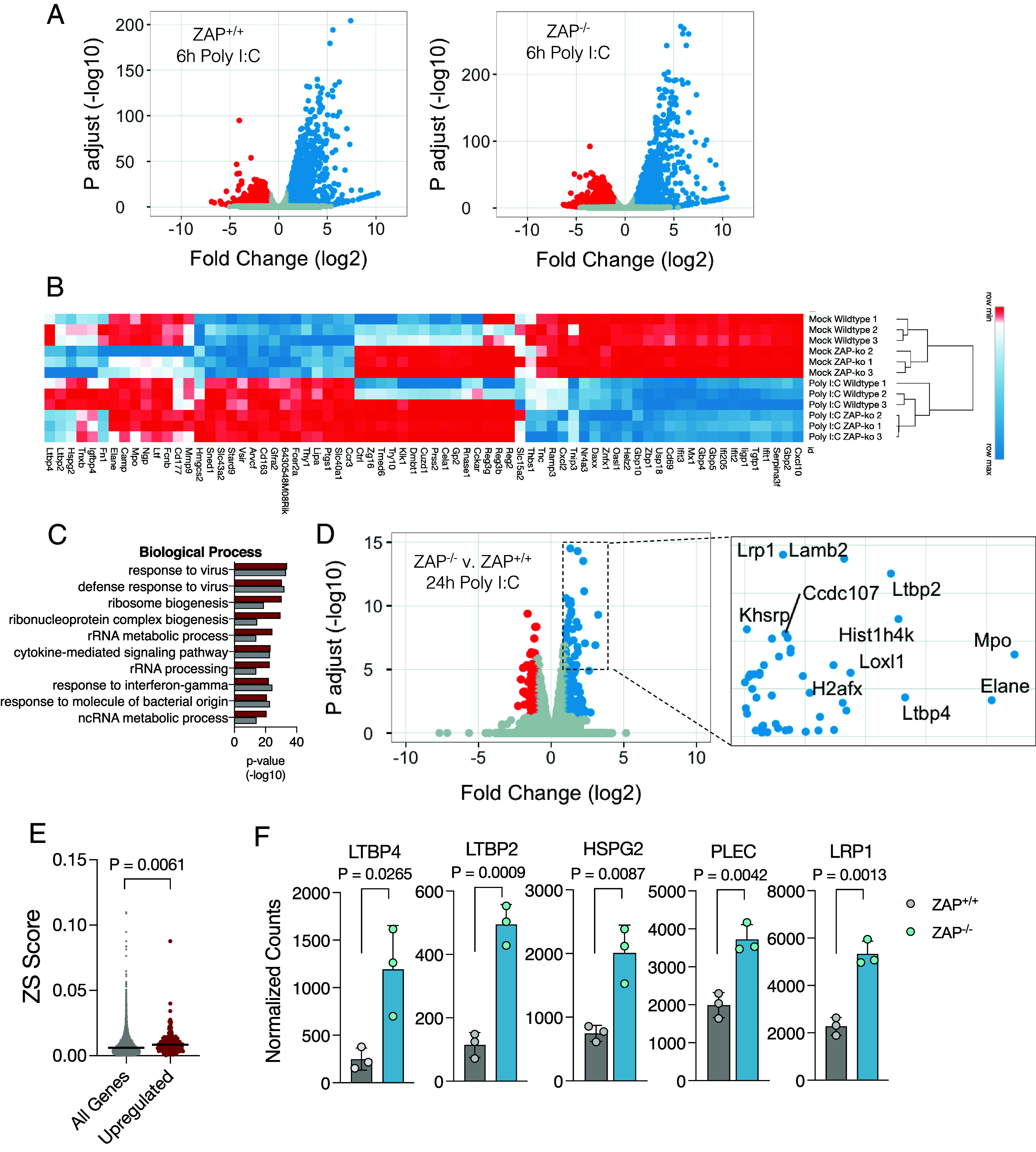
ZAP affects host gene expression at later stages of the innate immune response. (*A*) Transcriptomic changes in spleens from ZAP^+/+^ or ZAP^−/−^ mice 6 h after treatment with poly I:C (N = 3 per group). Blue dots indicate genes significantly upregulated upon poly I:C treatment and red dots indicate significantly downregulated genes. (*B*) Heatmap of the most upregulated (blue) or downregulated (red) genes in ZAP^+/+^ and ZAP^−/−^ mice treated with or without poly I:C. (*C*) Pathway analysis of the top differently expressed genes in response to poly I:C in ZAP^+/+^ and ZAP^−/−^ mice. (*D*) Differentially expressed genes in spleens from ZAP^+/+^ or ZAP^−/−^ mice 24 h after treatment with poly I:C (N = 3 per group). Blue dots indicate significantly increased genes and red dots indicate significantly decreased genes in ZAP^−/−^ mice compared to ZAP^+/+^ mice. *Inset* shows names of the most differently expressed genes in ZAP^−/−^ mice. (*E*) ZS score of all mouse genes versus genes with increased expression in ZAP^−/−^ mice at 24 h after treatment with poly I:C. (*F*) Expression (normalized counts) of *LTBP4*, *LTBP2*, *HSGP2*, *PLEC,* and *LRP1* in spleens from ZAP^+/+^ or ZAP^−/−^ mice 24 h after treatment with poly I:C. P, *P*-value.

Since ZAP is induced by stimulation with poly I:C (*SI Appendix*, Fig. S5*C*), its effects in gene expression might be exaggerated during an innate immune response and perhaps with kinetics that are delayed relative to IFN induction. To assess the impact of ZAP on host gene expression following poly I:C stimulus, we first compared gene expression in ZAP^+/+^ and ZAP^−/−^ mice at an early time point (6 h) after poly I:C injection. ZAP^+/+^ and ZAP^−/−^ mouse lines showed similar responses and most of the genes that were upregulated or downregulated in response to poly I:C were the same in the two lines (Fig. 5*A*). Hierarchical clustering and pathway analyses showed that most genes that were upregulated upon poly I:C treatment were involved in biological processes relating to defense against viruses, and many were canonical ISGs, as expected (Fig. 5 *B* and *C*). Poly I:C-treatment also induced downregulation of some genes in a manner that was similar in both ZAP^+/+^ and ZAP^−/−^ mice. Poly I:C-downregulated genes were primarily involved in signal transduction processes (*SI Appendix*, Fig. S6*A*). As previously observed in untreated mice, genes that are more abundantly expressed in ZAP^−/−^ mice than in ZAP^+/+^ mice included *LTBP4*, *LTBP2,* and *HSPG2* (Figs. 2*A* and 5 *A* and *B*). These data suggest that, overall, ZAP has little effect on gene expression at early time points after innate immune stimulation, beyond that seen at basal conditions.

Even though ZAP mRNA was increased in multiple organs 6 h after poly I:C administration (*SI Appendix*, Fig. S5*C*), differences in protein levels and downstream effects on ZAP target genes might be expected to occur at later timepoints. Therefore, we compared transcriptomes of spleens from ZAP^+/+^ and ZAP^−/−^ mice at 24 h after poly I:C injection (Fig. 5*D*). Genes that were more abundantly expressed in ZAP^−/−^ mice at this timepoint after poly I:C injection had higher ZS scores (Fig. 5*E*). Most genes whose expression was higher in ZAP^−/−^ mice than in ZAP^+/+^ mice at this timepoint were components of the extracellular matrix or had DNA-binding properties including nucleosome components, similar to the genes that were highly expressed in ZAP^−/−^ mice under resting conditions (Fig. 5*D* and *SI Appendix*, Fig. S6 *B* and *C*). Strikingly, some of the genes with high ZS scores, such as *LTBP2*, *LTBP4*, and *LRP1*, exhibited lower expression levels in ZAP^+/+^ mice compared to ZAP^−/−^ mice at 24 h after poly I:C injection. Conversely, in ZAP^−/−^ mice, these genes were either unaffected or showed increased expression levels (Fig. 5*F* and *SI Appendix*, Fig. S6*D*).

A possible confounding explanation for differences in mRNA abundance in the spleens of ZAP^+/+^ and ZAP^−/−^ mice is changes in the cellular composition of the spleen; therefore, we compared the levels of cell markers for T cells, B cells, monocytes, and granulocytes in RNA seq datasets (*SI Appendix*, Fig. S7 *A*–*D*). While there were some apparent fluctuations in splenocyte cell markers in response to poly I:C treatment, no significant differences were observed between ZAP^−/−^ and ZAP^+/+^ mice at 12 or 24 h post-injection and fluctuations in cell markers did not correlate with changes in ZAP-regulated genes. One exception was CD4 transcript levels that appeared lower in ZAP^−/−^ mice, when compared to ZAP^+/+^ mice. Overall, we conclude that later in the establishment of the innate immune response, following induction of ZAP, the expression of a subset of genes with high ZS scores is downregulated in a ZAP-dependent manner.

## Discussion

Previous work suggested that ZAP had evolved to exploit the paucity of CpG dinucleotides in the human genome to discriminate nonself from self RNA (6). Consistent with this idea, the scarcity of CpG dinucleotides is accentuated in genes responding to biological states where the expression of ZAP is induced (29). ZAP has been previously reported to regulate the expression of TRAILR4 in HeLa cells (21) as well as several IRGs (29), suggesting that the effects of ZAP on RNA stability are also observed in endogenous genes. However, predicting which genes are targeted by ZAP solely based on CpG dinucleotide frequency is difficult, and this parameter is not the sole determinant of sensitivity to ZAP (23). Based on our previous finding that the number of CpG dinucleotides, distance between CpG dinucleotides, and the mononucleotide composition of the surrounding sequence contribute to the antiviral activity of ZAP in HIV-1 and enterovirus A71 (23), we developed a scoring method that quantifies and integrates these features to generate a combined ZS score for all ORFs. Our finding that most human and mouse genes have very low ZS scores, indicates that not only the low abundance of CpGs, but also their suboptimal spacing and surrounding composition of the sequences surrounding these CpGs make the majority of genes poor ZAP targets.

It has been previously reported that the rate of spontaneous, random deamination of methylated CpGs in DNA is insufficient to explain the extent to which coding sequences have been purged of CpG dinucleotides (37). Additional selection pressures may therefore exist that shaped the overall nucleotide composition of host mRNA. Our data are consistent with the idea that ZAP may have contributed for this selection, particularly since both human and mouse—two species where ZAP is present—showed similar CpG distribution patterns and ZS score distributions, which differ from those of drosophila genomes (where ZAP is absent). However, drosophila genomes also have much lower levels of methylated DNA compared to human genomes—probably due to the lack of expression of DNA methyltransferases (38)—suggesting that CpG suppression may have been driven by a combination of CpG methylation/deamination and selection through ZAP activity. Further evidence is necessary to determine whether the presence of ZAP has contributed to the paucity and distribution of CpG dinucleotides in mammalian genes.

Despite the low ZS scores of most human genes, a small group of human transcripts had comparatively high ZS scores, as did their orthologues in the mouse genome. The expression of most of these genes was increased in ZAP^−/−^ mice and in ZAP-deficient human T cell lines, strongly suggesting that their expression is modulated by ZAP in a manner that is conserved across these species. While we did not calculate ZS scores for other species, specific endogenous ZAP targets should exist in other mammals since their ZAP proteins have similar antiviral activity and their genomes exhibit similar levels of genomic CpG suppression (20). Nevertheless, antiviral activity and target specificity for most species have not been investigated; thus, cross-species comparisons should be made with caution. Similarly, the parameters of this algorithm may be adjusted as more insight into the biology of ZAP is revealed.

Most ZAP regulated genes were either involved in nucleosome assembly or in the organization of the extracellular matrix. One notable ZAP-regulated gene encodes HSPG2, also known as perlecan, which is an integral component of the extracellular matrix (39). HSPG2 binds growth factors and connects proteins in the plasma membrane with fibers of the extracellular matrix, such as collagen and laminin (40), and it has been suggested to play a role in numerous diseases including atherosclerosis (41), cancer metastasis (42) and Alzheimer’s disease (43). Ablation of HSPG2 in mice causes embryonic lethality, due to defects in the myocardial basement membrane (44). HSPG2 mutations in humans cause dyssegmental dysplasia, a lethal autosomal recessive disorder accompanied by short-limbed dwarfism (45), as well as nonlethal chondrodysplasia and early onset osteoarthritis (46). High HSPG2 expression levels negatively correlate with the survival of patients suffering from acute myeloid leukemia (47). Nonetheless, we did not observe any obvious defects in ZAP^−/−^ mice – with the exception of increased EV-A71 disease burden (23). Our model provides a useful tool to study a potential role of changes in ZAP target genes such as HSPG2 in sensitization to disease.

A curious observation was the prolonged expression of IFN transcripts in ZAP^−/−^ mice after treatment with poly I:C. This finding is in agreement with a previous report that made similar observations in cell lines (31). This finding is superficially surprising because IFN genes show low frequencies of CpG dinucleotides and low ZS scores. Schwerk and colleagues proposed that ZAP interacts with IFN transcripts by directly binding to AUUUA motifs in the 3′ UTRs While we previously observed that a sequence surrounding a CpG dinucleotide rich in adenosine and/or uridine enhances ZAP’s activity, both CLIP-Seq and crystallographic approaches do not provide evidence for a direct interaction between ZAP and AUUUA motifs (6, 11, 12). The effect of the absence of ZAP on the prolonged expression of IFN transcripts may be a consequence of altered chromatin states (48). Our data show that ZAP targets and downregulates mRNAs for histones and transcription factors, which may indirectly modulate the expression of interferon genes and proinflammatory molecules. In ZAP-deficient mice, there is a notable upregulation of transcriptional repressors and a decrease in RNA-binding proteins that regulate inflammatory responses. While these studies point to an indirect role of ZAP in the modulation of IFN transcripts, further investigation is required to clarify precisely how this occurs.

Overall, we demonstrate that a subset of genes can be predicted and demonstrated to be endogenous ZAP targets in at least two mammalian species. These findings provide a tool that can be used to predict ZAP targets and provide a model and insight to study the effects of this antiviral protein in modulating host gene expression.

## Methods

### Cells.

Human embryonic kidney (HEK) 293 T cells were cultured in Dulbecco’s modified Eagle medium (Gibco, Thermo Fisher) supplemented with 10% fetal bovine serum (FBS) and gentamicin. Human T lymphocyte cell lines MT4 and SupT1 were cultured in RPMI (Gibco, Thermo Fisher) and supplemented with 10% FBS and gentamicin. All cell lines were kept at 37˚C and 5% CO_2_.

### Animals.

C57BL/6 and C57BL/6 ZAP^−/−^ mice were housed as previously described (23). All experiments in which these animals were used were conducted in accordance with guidelines of The Rockefeller University Institutional Animal Care and Use Committee.

A total of 24 female and 12 male 9-wk-old mice were used for this study. Sex of the mice was matched in each group. Three mice (one male and two females) were used per group. Polyinosinic–polycytidylic acid (poly I:C, Sigma) was dissolved in molecular biology grade water and filtered through a 0.45 µm PVDF filter.

Poly I:C-treated mice were injected intraperitoneally with a solution of 5% glucose, jetPEI transfection reagent (PolyPlus), and 10µL of a 10 mg/mL poly I:C solution. Mock-treated mice were injected with a solution of 5% glucose and jetPEI transfection reagent. At 6, 12, and 24 h after administration of poly I:C or mock solution, mice were killed and organs (spleen, heart, lung, thymus, brain, kidney, and liver) were immediately harvested, washed in cold PBS and submerged into RNAlater preservation solution (Thermo Fisher). Samples were then kept at -80˚C until further use.

### RNA Extraction.

For animal samples, organs were thawed and homogenized in a solution of TRIzol reagent (Thermo Fisher) and ultrapure molecular biology grade water (3:1 ratio) using an Omni TH Hand-held Homogenizer (Camlab, UK) as previously described (23). RNA pellets were resuspended in 200µL of ultrapure water and frozen at -80˚C until further use. For samples generated from cell lines, cells were lysed in RA1 buffer containing β-mercaptoethanol and RNA was extracted using the NucleoSpin RNA Mini kit (Macherey-Nagel).

### Quantitative PCR.

RNA concentration from samples of the same experiment was measured by spectrophotometry and equalized across all samples. Complementary DNA (cDNA) was synthesized using 11µL of total RNA were used for complementary DNA production with a SuperScript IV First-Strand Synthesis System (Thermo Fisher). Two microliters of cDNA were then used in TaqMan Gene Expression assay (Thermo Fisher) according to the manufacturer’s guidelines. The following qPCR probes were purchased from Thermo Fisher: mouse IFNA1/5/6 (Mm03030145_gH), mouse IFNB1 (Mm00439552_s1), mouse IFNG (Mm01168134_m1), mouse IFNL3 (Mm00663660_g1), mouse ISG15 (Mm01705338_s1), mouse ZC3HAV1 (Mm00512227_m1), mouse ACTB (Mm02619580_g1), human ISG15 (Hs01921425_s1), human ZC3HAV1 (Hs00912660_m1), human IFNA1 (Hs00265051_s1), human IFNB1 (Hs01077958_s1), human IFNG (Hs00989291_m1), (Hs00820125_g1) human IFNL2, (Hs02786624_g1) human GAPDH, (Hs01078482_g1) human HSPG2, (Hs00943217_m1) human LTBP4 and (Hs00166367_m1) human LTBP2.

### RNA Sequencing and Bioinformatic Analysis.

Total RNA samples were submitted to Novogene Co., LTD (China), and prepared for RNA sequencing. mRNA was enriched by removal of ribosomal RNA, followed by cDNA synthesis through reverse transcription. Samples were sequenced in an Illumina NovoSeq 6000 paired-end 150. Raw reads were aligned to the mouse genome (*Mus musculus* ensemble 94) using STAR aligner and differentially gene expression analysis was performed using DESeq2 (49). Differentially expressed genes were considered such using a log2-fold change threshold of >1.5 or <−1.5 and p-adjusted value smaller than 0.05. Network and enrichment analysis was conducted using STRING(50). Data were plotted using the ggplot2 package for R (https://cran.r-project.org/web/packages/ggplot2/index.html). All RNA-Seq data acquired and processed in this study can be found on NCBI GEO under the accession number (GSE274578 and GSE274582).

### In Silico Analysis of ZAP Sensitivity in Coding Sequences.

In silico libraries of open reading frames were generated by retrieving cDNA or mRNA transcript sequences from Ensembl (*Homo sapiens*, release 99; *Mus musculus* c57bl6nj, release 99; *D. melanogaster*, release 108). Sequences were imported and processed in R using in-house-built scripts (available at https://github.com/DanSallves/ZAPbind). Briefly, these scripts take as input a fasta file containing DNA sequences to be analyzed and calculate ZS scores based in three metrics:

(i) CG scores were calculated by determining the frequency of CpG dinucleotides in a given sequence as a fraction of the total number of dinucleotides. normalized to the maximum CpG frequency detected (= 0.173).
(ii) Distance scores were generated by first computing total number of optimally spaced CpG dinucleotides (12 to 32 nucleotides apart) for each gene (total CG pairs score) then the Distance scores was calculated as the fraction of optimally spaced CpG in relation to the total number of CpG pairs for each gene.
(iii) Composition scores were generated by first calculating the fraction of adenosine and thymine nucleotides in the sequences between each CpG pair that was spaced 12 to 32 nucleotides apart. Then, for each transcript, the mean composition for each optimally placed CpG pair was calculated.

The CG score, Distance score, and Composition score were normalized to a value between 0 and 1, by dividing the computed score for each transcript by the maximum score observed for each metric. ZAP-sensitivity scores were then calculated as the product of these three normalized scores (CG score, Distance score and Composition score) for each sequence. Data were then visualized using the ggplot2 in R, and statistical significance was inferred using R. Enrichment and network analysis was performed using STRING (50). Network plots were produced using Cytoscape (51).

### RNA Interference.

Cells were washed in PBS and gently pelleted in 1.5 mL centrifuge tube at 90×*g* for 10 min at room temperature. Cells were then resuspended in P3 primary cell nucleofector and supplement solution (Lonza) and 500 µmol of an siRNA pool targeting ZAP or a nontargeting control (NTC, Dharmacon). Cells were then transferred to a nucleocuvette and electroporated twice using a 4D-Nucleofector X Unit (Lonza). Cells were allowed to rest for 10 min at room temperature and were then transferred to a 12-well dish containing complete RPMI. Cells were incubated for 48 h at 37˚C prior to analysis.

### CLIP–Seq.

RNA:protein complexes were isolated and sequenced as previously described (52). In brief, MT4 cells were transduced with a doxycycline-inducible construct encoding for the long isoform of ZAP with three HA tags at the C-terminus (3xHA). Cells were grown in complete medium containing 4-thiouridine overnight; RNA and proteins were then cross-linked at 0.15 J cm^−2^ UV (λ = 365 nm) in a Stratalinker 2400 UV crosslinker (Stratagene). ZAP:RNA complexes were immunopurified using Protein G-conjugated magnetic Dynabeads and a mouse monoclonal anti-HA antibody, and the RNA was radiolabeled with ATP-γ-32P. Protein–RNA adducts were resolved by SDS–PAGE, transferred to nitrocellulose, and detected by autoradiography. Sequential 3′ and 5′ adaptor ligations were performed, as described before (52), followed by reverse transcription. Sequencing of the cDNA library was performed on an Illumina HiSeq 2000 platform. Sequencing data were processed as previously described (52, 53), using the FASTX toolkit (https://github.com/Debian/fastx-toolkit), excluding reads with fewer than 15 nucleotides. Reads were then aligned to the human genome (hg39). For comparison of ZAP binding to high ZS score and low ZS score genes, a group of 17 high ZS score transcripts was matched to low ZS score transcripts that had approximately the same transcript length (±2,000 bp) and approximately equivalent expression (±1 FPKM). The matched gene with the lowest ZS score was selected and CLIP-seq reads for high ZS score and low ZS score genes enumerated. Normalized reads were calculated by dividing total reads by transcript length.

### Western Blot Analysis.

Cells were lysed in NuPAGE buffer containing β-mercaptoethanol and protein lysates were resolved onto a 4 to 12% NuPAGE polyacrylamide gel (Thermo Fisher). Proteins were then transferred to a nitrocellulose membrane and blocked in 5% milk PBS-Tween20 or blocking buffer (Licor). Rabbit anti-ZAP (1:5,000, 16820-1-AP, Protein Tech) and anti-tubulin (1:10,000, T5168, Millipore Sigma) antibodies were incubated overnight at 4˚C. Membranes were then washed in PBS-Tween20 and incubated with secondary antibodies either conjugated with horseradish peroxidase and developed using a C-Digit chemiluminescent scanner or conjugated with IRDye and developed on an Odyssey Licor scanner.

### Statistical Analysis.

Analysis of the distribution of CG scores, Distance scores, and Compositions scores among coding sequences from the human, mouse, or fly were performed using the Welch *t* test using R. Unless stated otherwise, unpaired Student’s *t* tests were performed to infer statistical significance using GraphPad Prism; *P*-values and false discovery rates (FDR, q-value) are present in relevant graphs.

## Supplementary Material

Appendix 01 (PDF)

## Data Availability

RNA seq data have been deposited in NCBI GEO (GSE274578 and GSE274582) (54, 55). All other data are included in the manuscript and/or *SI Appendix*.
