# Supplementary material for: Modulation of host gene expression by the zinc finger antiviral protein
Source: Proc Natl Acad Sci U S A. 2025 Mar 27;122(13):e2420819122. doi: 10.1073/pnas.2420819122 (PMC12002351; doi:10.1073/pnas.2420819122)
Supplement: Supplementary file 1 — Appendix 01 (PDF) [file pnas.2420819122.sapp.pdf]

Supplemental information
Supplemental Figures 1-7

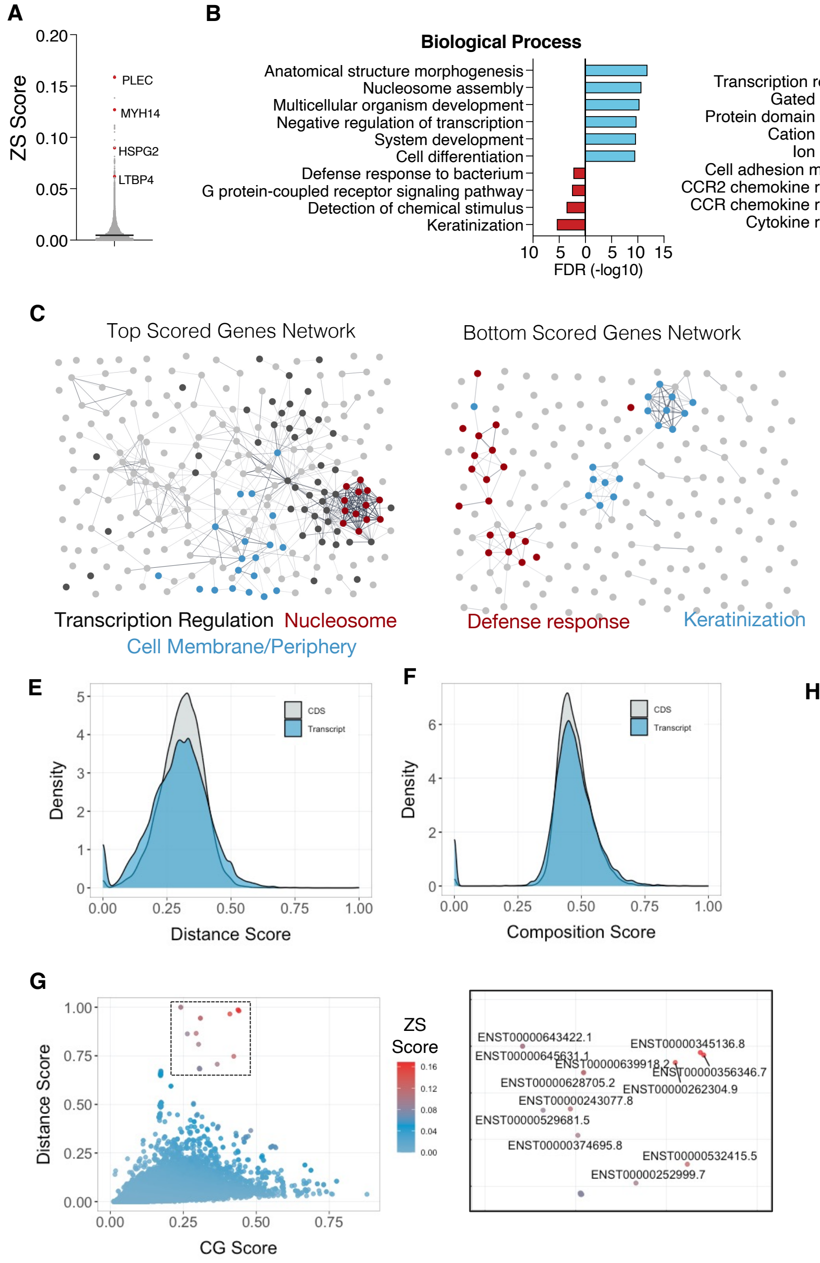

Transcript ID	Gene
ENST00000243077.8	LRP1
ENST00000252999.7	LAMA5
ENST00000262304.9	PKD1
ENST00000345136.8	PLEC
ENST00000356346.7	PLEC
ENST00000374695.8	HSPG2
ENST00000529681.5	MUC5B
ENST00000532415.5	PKD1P5
ENST00000628705.2	FCGBP
ENST00000639918.2	FCGBP
ENST00000643422.1	MUC2
ENST00000645631.1	MUC2

Supplemental Figure 1 – ZAP sensitivity scores in protein coding and full transcript sequences.
(A) Distribution of ZS scores in human protein coding sequencing with highly scored genes highlighted. (B) Gene enrichment analysis of highly scored (blue) and poorly scored (red) genes based on biological process and molecular function. (C) Network analysis of the top and bottom scored human coding sequences. (D-F) Distribution of CG scores (D), distance scores (E) and composition scores (F) comparing human coding sequences (CDS, grey) and mRNA transcript sequences (blue). (G) Distribution of ZAP-sensitivity (ZS) scores among human full length transcript sequences. Expanded inset indicates transcript IDs of highly scored genes (H) Proteins encoded by high ZS score full length transcripts.

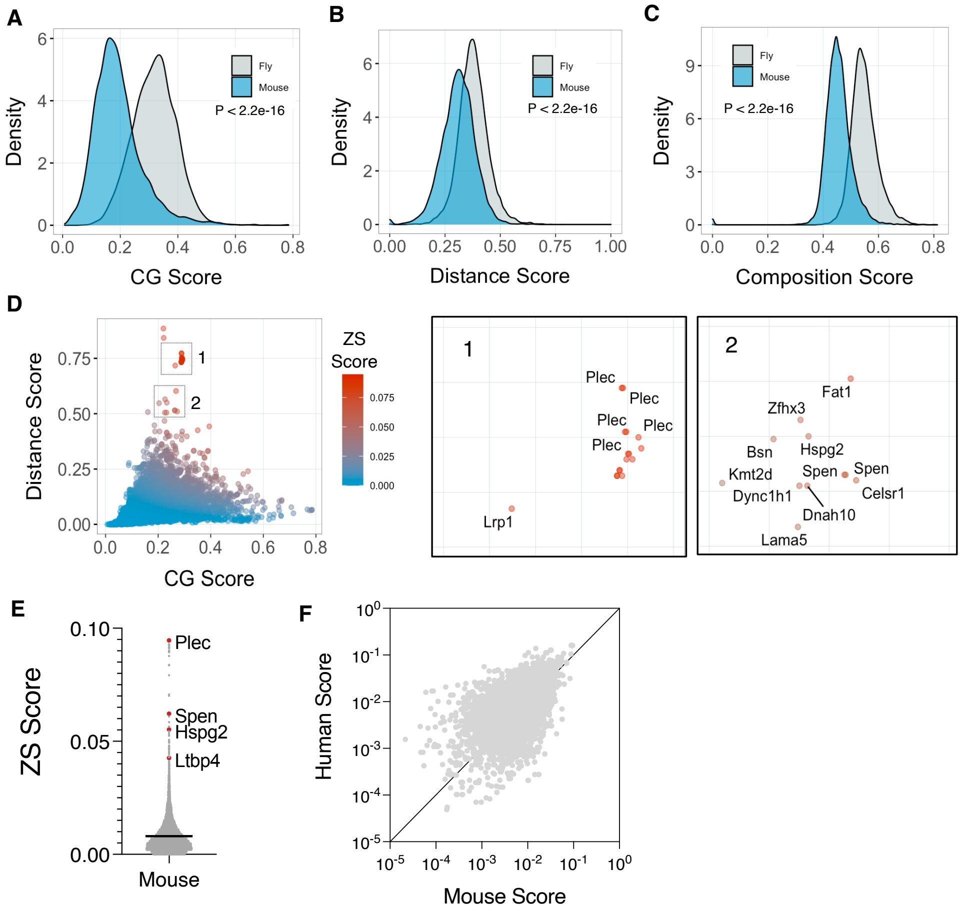

Supplemental Figure 2 – Comparable ZAP sensitivity scores in human and mouse coding sequences.

(A-C) Distribution of CG scores (A), distance scores (B) and composition scores (C) in coding sequences comparing mouse (Mus musculus) – and fly. (D) Distribution of ZS scores among mouse coding sequences. Insets 1 and 2 indicate groups of highly scored genes. (E) Distribution of ZS scores in mice with highly scored genes highlighted. (F). Correlation between ZS scores of orthologous sequences in human and mice. P, p-value.

A

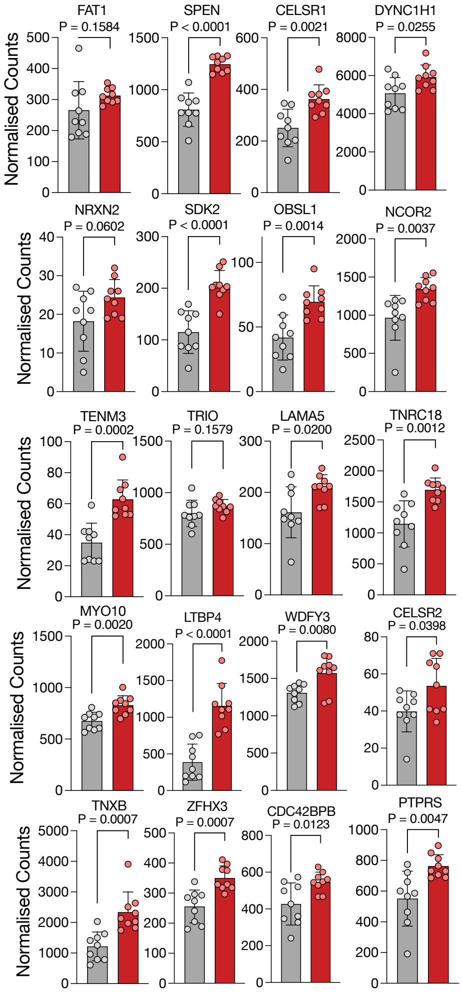

B

	Score	Upregulated in ZAP-/-
Plec	0.095	Yes
Lrp1	0.088	Yes
Fat1	0.084	No
Spen	0.062	Yes
Celsr1	0.061	Yes
Dync1h1	0.056	Yes
Zfhx3	0.056	Yes
Hspg2	0.055	Yes
Nrxn2	0.054	No
Sdk2	0.053	Yes
Obsl1	0.051	Yes
Ncor2	0.050	Yes
Ptprs	0.050	Yes
Tenm3	0.050	Yes
Trio	0.047	No
Lama5	0.046	Yes
Tnrc18	0.044	Yes
Cdc42bpb	0.043	Yes
Myo10	0.043	Yes
Ltbp4	0.043	Yes
Wdfy3	0.043	Yes
Celsr2	0.041	Yes
Tnxb	0.040	Yes

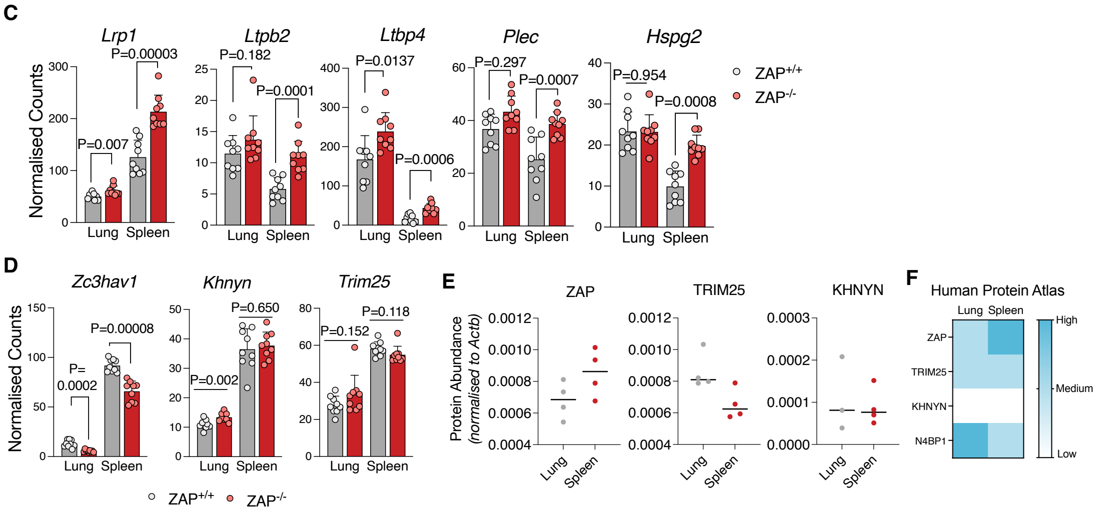

Supplemental Figure 3 – Expression of high ZS-score genes is increased in ZAP-/- mice.

(A) Normalized counts of highly scored genes in mock-treated ZAP+/+ and ZAP-/- mice (N = 9). (B) List of high ZS score mouse coding sequences (top 0.1% of ZS scores). (C,D) Normalized counts (FPKM) of highly scored genes (C) and ZC3HAV1, KHNYN and TRIM25 (D) in lung and spleen samples from wildtype and ZAP-deficient mice. (E) Protein abundance relative to actin-beta (Actb) in mouse lungs and spleens in Lu et al. 2022. (F) Protein abundance in histological samples of human lungs and spleens in the Human Protein Atlas database. P, p-value.

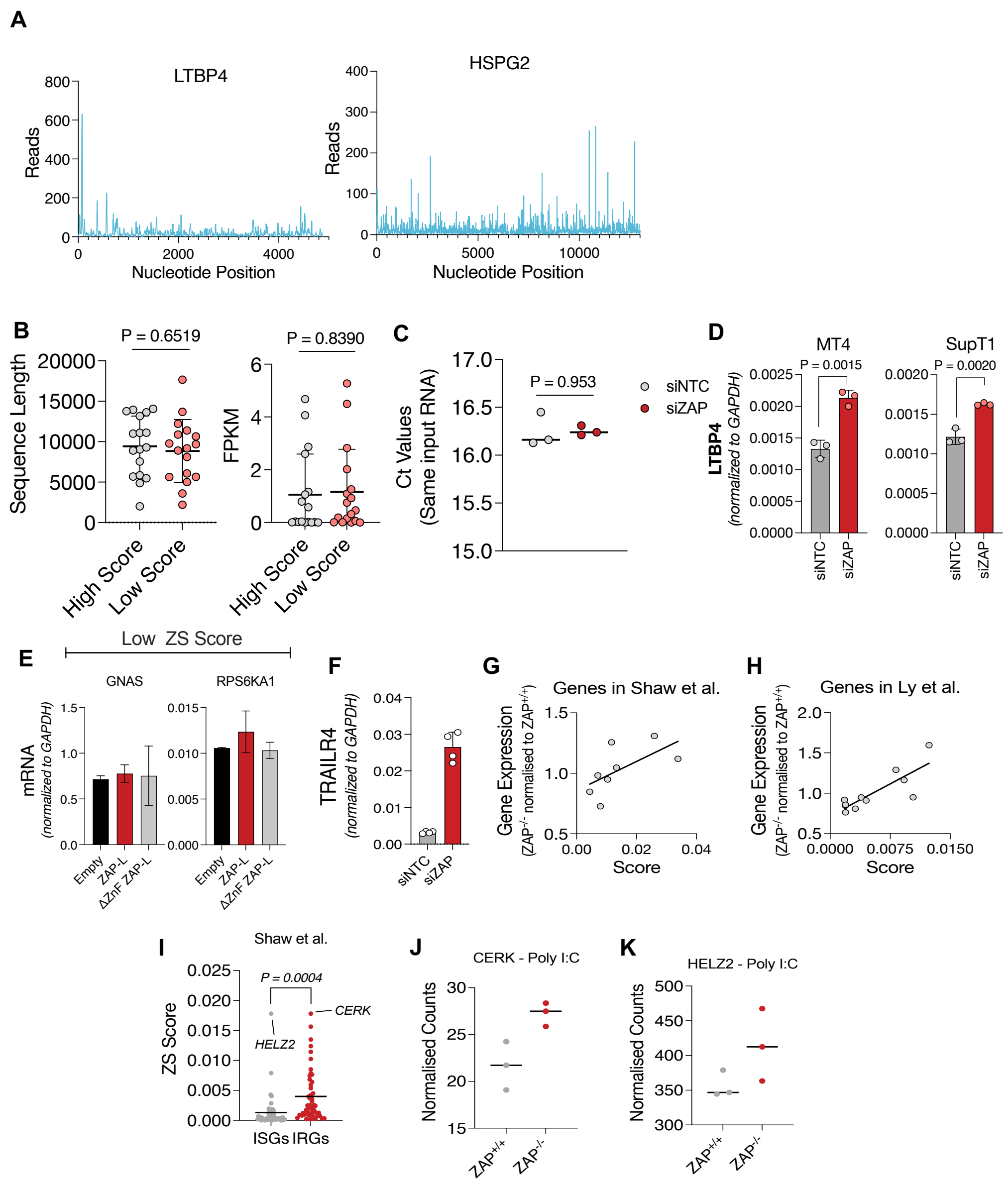

Supplemental Figure 4 – Gene expression regulation and ZAP-mRNA binding in human cells.

(A) CLIP-seq analysis of ZAP binding to LTBP4 and HSPG2 mRNAs in MT4 cells. The number of reads spanning each nucleotide is plotted against nucleotide position.
(B) Quantification of sequence length (left), transcript abundance (right) based on RNA-Seq experiments for high ZS score and low ZS score genes expressed in MT4 cells. (C) CT values for *GAPDH* in cells treated with non-targeting or ZAP-targeting siRNAs. (D) Expression of LTBP4 measured using qPCR in human T cell lines (MT4 and SupT1) transfected with siRNAs targeting ZAP or a non-target control (NTC). (E) Expression of *GNAS*, *RPS6KA1*, *TNFRSF10D* and *SPEN* in ZAP-knockout cells reconstituted with an empty vector, ZAP-L or a truncated form of ZAP-L lacking the RNA-binding domain (ΔZnF ZAP-L). (F) Expression of *TNFRSF10D (TRAILR4)* in cells treated with non-targeting (NTC) or ZAP-targeting siRNAs. (G-H) Correlation between ZS scores and fold-change in expression between $ZAP^{-/-}$ and $ZAP^{+/+}$ mice in genes identified in Shaw et al. and Ly et al.
(I) ZS scores of interferon-stimulated genes (ISGs) and interferon-repressed genes (IRGs).
(J) Expression of *CERK* and *HELZ2* in $ZAP^{-/-}$ and $ZAP^{+/+}$ mice 6h after treatment with Poly I:C). P, p-value.

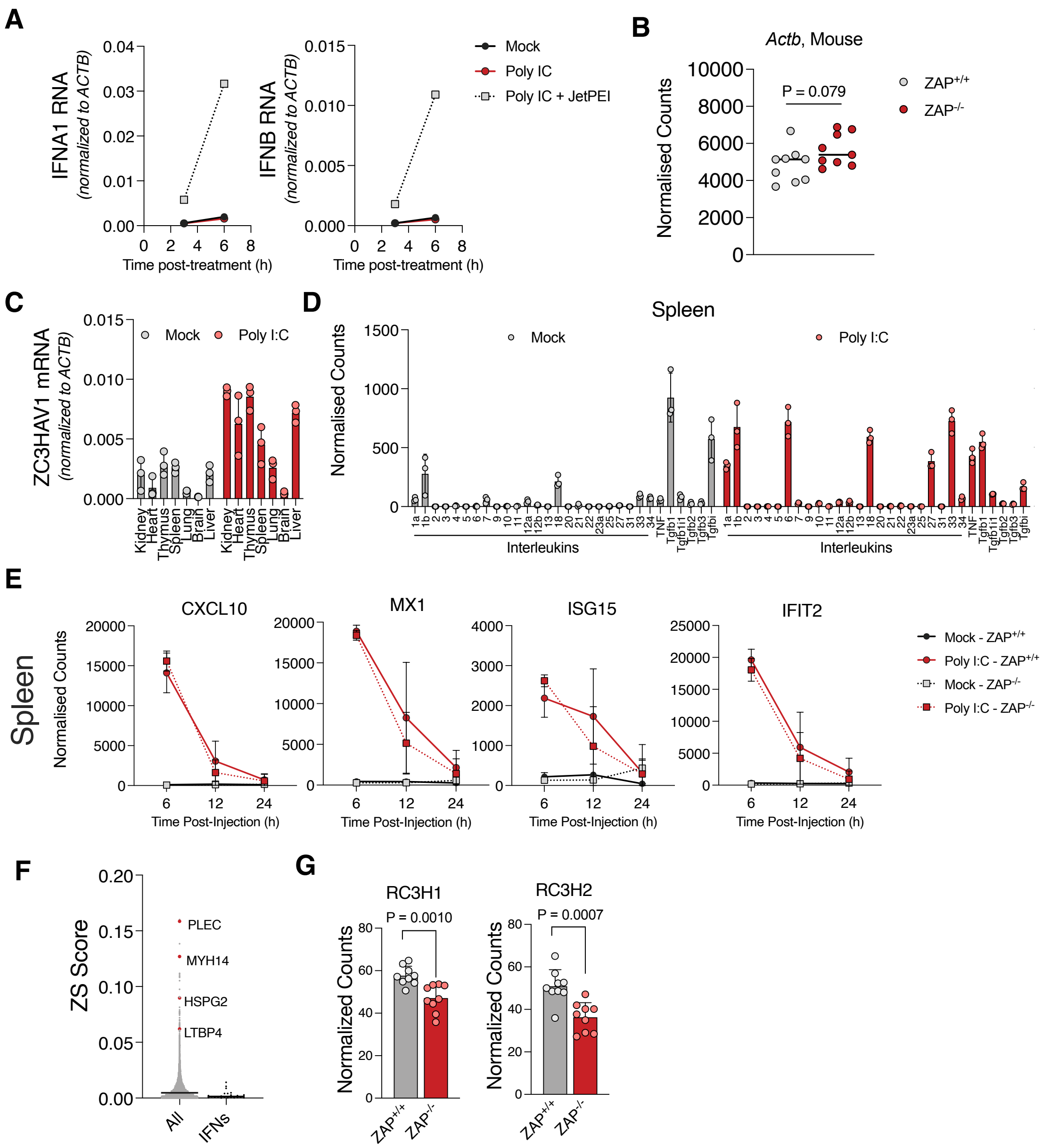

Supplemental Figure 5 – Transcriptomics changes in mice at 6h after poly I:C treatment.

IFNA and IFNB expression measured by qPCR in ZAP+/+ mice injected intraperitoneally with poly I:C conjugated with or without a transfection reagent (JetPEI). Spleens were harvested 2 or 6h post-injection (N = 2). (B) Expression levels of ACTB in ZAP$^{+/+}$ and ZAP$^{-/-}$ mice. (C) ZAP/ZC3HAV1 expression in indicated organs of ZAP+/+ mice 6h after injection with poly I:C (N = 3). (D) Expression (normalized counts) of indicated cytokines detected by RNA-seq in spleens of ZAP+/+ mice 6h after injection with poly I:C. (E) Expression (normalized counts) of CXCL10, MX1, ISG15 and IFT2 in spleens from ZAP+/+ and ZAP-/- mice treated with poly I:C. (F) Comparison of ZS scores of mouse coding sequences and mouse IFN transcripts. (G) Expression (normalized counts) of RC3H1 and RC3H2 genes in spleens from ZAP+/+ or ZAP-/- mice.

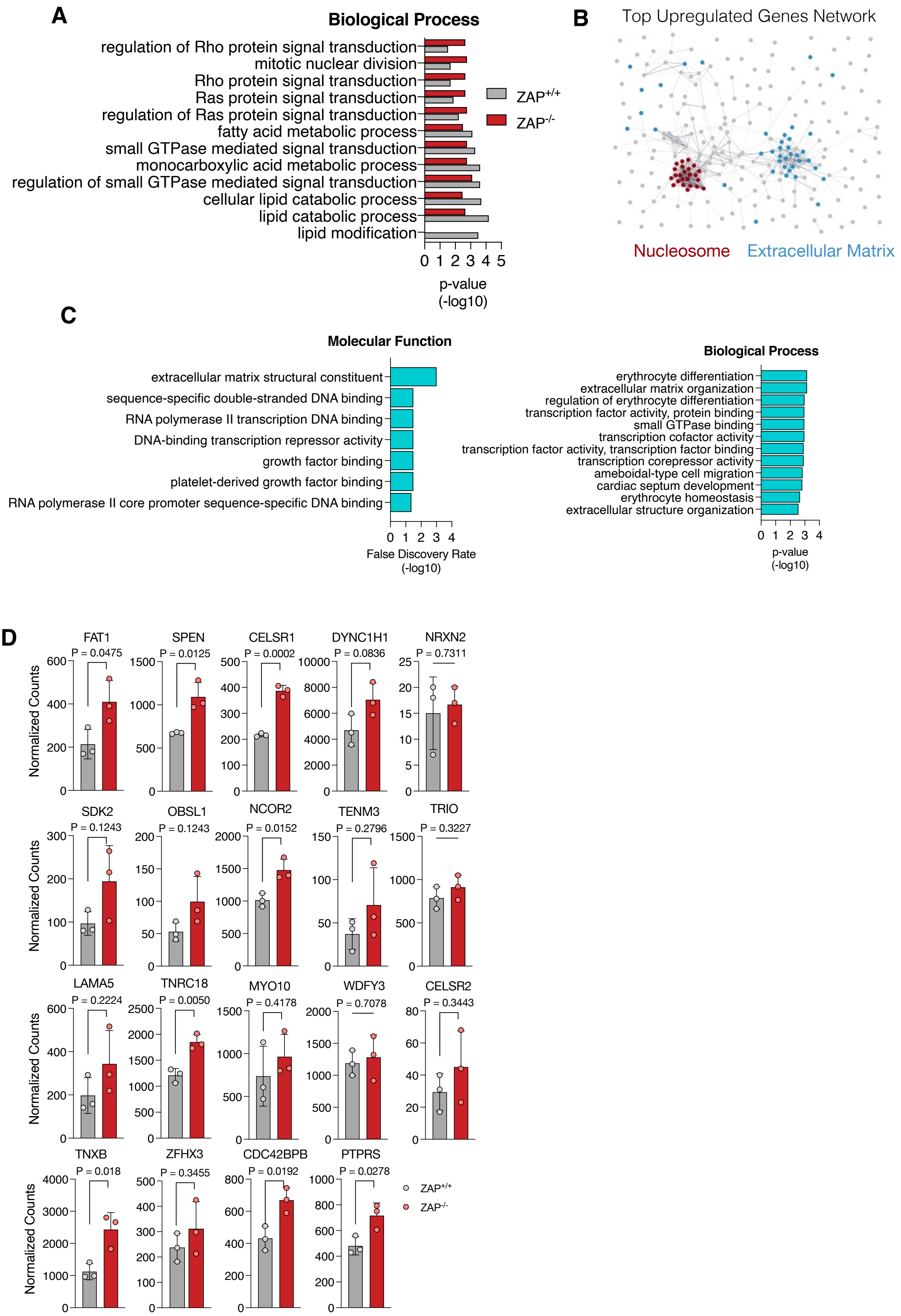

Supplemental Figure 6 – Transcriptomic changes in mouse spleens after 24h treatment with poly I:C.

(A-C) Pathway and network analyses of differently expressed genes in ZAP-/- mice 24h after treatment with poly I:C. (D) Normalized counts of top scored genes in spleens from ZAP+/+ or ZAP-/- mice 24h after treatment with poly I:C. P, p-value.

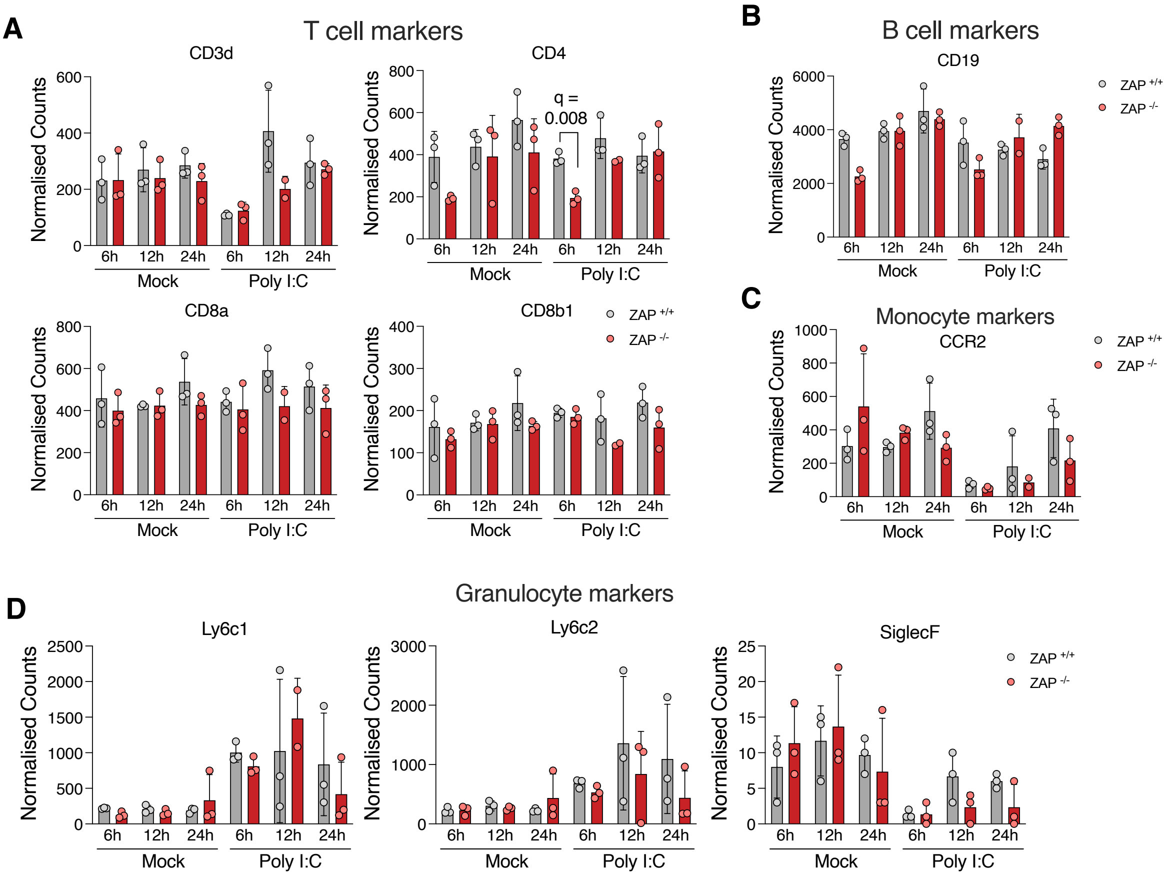

Supplemental Figure 7 – Expression of immune cell markers in mouse spleens. (A-D) Expression (normalized counts) of T cell (A), B cell (B), monocyte (C) and granulocyte (D) surface cell markers in RNA samples extracted from spleens of ZAP+/+ and ZAP-/- mock- or poly I:C-treated mice. Statistical q values are provided only when comparisons are statistically significantly different. Unstated comparisons are non-significant.
